# Cold Tolerance of the Male Gametophyte during Germination and Tube Growth Depends on the Flowering Time

**DOI:** 10.3390/plants6010002

**Published:** 2016-12-29

**Authors:** Johanna Wagner, Evelyn Gastl, Martin Kogler, Michaela Scheiber

**Affiliations:** Institute of Botany, Faculty of Biology, University of Innsbruck, Sternwartestrasse 15, 6020 Innsbruck, Austria; evelyn.gastl@hotmail.com (E.G.); martin.kogler90@gmail.com (M.K.); michaela.scheiber@hotmail.com (M.S.)

**Keywords:** freezing tolerance, in vitro, plant reproduction, pollen germination, pollen tube growth, sub-zero temperature

## Abstract

In temperate climates, most plants flower during the warmer season of the year to avoid negative effects of low temperatures on reproduction. Nevertheless, few species bloom in midwinter and early spring despite severe and frequent frosts at that time. This raises the question of adaption of sensible progamic processes such as pollen germination and pollen tube growth to low temperatures. The performance of the male gametophyte of 12 herbaceous lowland species flowering in different seasons was examined in vitro at different test temperatures using an easy to handle testing system. Additionally, the capacity to recover after the exposure to cold was checked. We found a clear relationship between cold tolerance of the activated male gametophyte and the flowering time. In most summer-flowering species, pollen germination stopped between 1 and 5 °C, whereas pollen of winter and early spring flowering species germinated even at temperatures below zero. Furthermore, germinating pollen was exceptionally frost tolerant in cold adapted plants, but suffered irreversible damage already from mild sub-zero temperatures in summer-flowering species. In conclusion, male gametophytes show a high adaptation potential to cold which might exceed that of female tissues. For an overall assessment of temperature limits for sexual reproduction it is therefore important to consider female functions as well.

## 1. Introduction

Among various environmental factors, temperature is one of the main factors shaping the geographical and elevational distribution of plants [1,2] and the temporal patterning of phenology [3]. To colonize a habitat over the long term all developmental phases must be adapted to the prevailing temperature conditions. Among the reproductive phases male gametogenesis (reviewed in [4]) and anthesis is particularly susceptible to unfavorable temperatures. During anthesis, cold and heat disturb male functions (pollen germination, pollen tube growth), female functions (receptivity of stigma, style, and ovules), and male-female interactions (pollen adhesion, pollen tube guidance, and fertilization) [5,6,7,8,9]. The failure of only one function can lead to a complete loss of the offspring [7,10].

The temperature range in which sexual processes normally operate varies among species and genotypes according to the climatic conditions in their habitat [5,11,12,13]. The cold limit for pollen germination and pollen tube growth was found above 10 °C in plants from warmer climates [14,15], and between 5 and 10 °C in most lowland plants in the temperate zone [13,16,17]. In high-mountain plants, which may experience sub-zero temperatures also during the growing period, reproductive processes such as pollen germination, pollen tube growth, and fertilization still operate around 0 °C [9,18]. In a recent study on a number of plant species growing along an elevational gradient, Rosbakh and Poschlod [13] confirmed the positive relationship between temperature conditions at the growing site at peak flowering and the minimum temperature for pollen germination and pollen tube growth. The authors concluded that the restriction of pollen performance at low temperatures contributes to the climatic restriction of plant species distribution along climatic gradients. A similar relationship between minimum temperatures during the flowering period and the cold limit for pollen activity can be expected in a temperate lowland climate in the course of a year. Plants flowering in winter and early spring are regularly exposed to frost which may even be more severe and long-lasting than during summer cold-spells in high-mountains [19]. Later in spring, sub-zero temperatures occur only occasionally, and summer flowering lowland species usually do not experience frost at all. As indicated above, patterns of flowering phenology in plant communities are the result of a long-term adaptation to environmental conditions [20]. Plant species that are not strictly controlled by day-length show a certain degree of phenological plasticity whereby onset and duration of flowering are primarily driven by temperature [21]. In the course of climate warming there is a trend towards an earlier beginning of the growing season [22]. However, an earlier start increases the probability that usually mid and late spring flowering species are more often exposed to intermittent cold periods and late frost events [3,23]. Depending on how well plants cope with low temperatures during the most susceptible reproductive phases, the reproductive output would be more or less impaired [24]. In the long term it can be expected that changes in the reproductive fitness lead to changes in the composition of plant communities [25,26]. 

In the present study we tested the hypothesis that the low temperature limit of susceptible anthesis processes correlates with the flowering time. We focused on the performance of the male gametophyte which offers the advantage to examine a greater number of species using an in vitro test system. On the basis of the protocol of Boavida and McCormick [27], we developed an easy to handle system for the exposure to different temperatures and for later microscopic examination. We determined the cold limits of pollen germination and pollen tube growth of some winter, spring, and summer flowering herbaceous plant species from the lowlands in Central Europe (Table 1). To test the pollen performance in the cold we exposed pollen on an agarose medium directly to different test temperatures and determined germination rates and the dynamics of pollen tube growth at different times. In a further step, we tested the frost tolerance of activated male gametophytes. Pollen was subjected to step cooling (cooling down, exposure to freezing temperatures, and warming-up), and checked for the capacity to resume pollen tube growth after the exposure to low temperatures. Since winter and early-spring flowering plants regularly have to cope with sub-zero night temperatures we expected pollen to perform at least as well as in high-mountain species in the cold. On the other hand, we assumed that summer flowering species would be less adapted to the cold than earlier flowering species. On the basis of the results, we estimated the adaptive capacity of the male gametophyte to low temperatures in individual species.

## 2. Results

**Figure 1:**
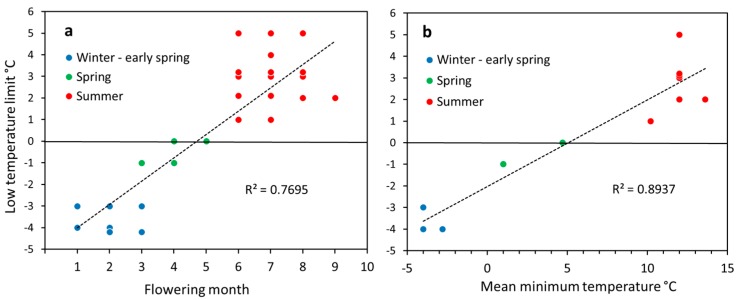
Relationship between low temperature limit of pollen germination (i.e., germination is zero or nearly zero) and (**a**) the flowering month and (**b**) the mean minimum air temperature in the coldest month of flowering. Symbols in (**a**) represent the temperature limits for the species flowering in the respective months. Dotted line: linear regression line.

**Figure 2:**
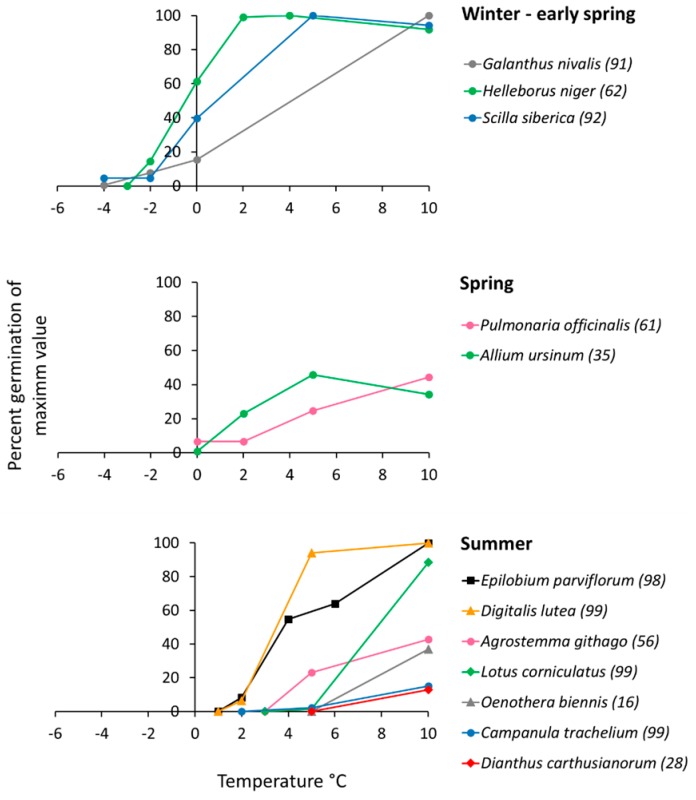
Pollen germination in the different phenotypes within 24 h in relation to temperature. For comparability among species, germination values are given in percent of the respective control value (=100%) in the optimum temperature range. Species specific control percentage is given in brackets next to the species name.

**Figure 3:**
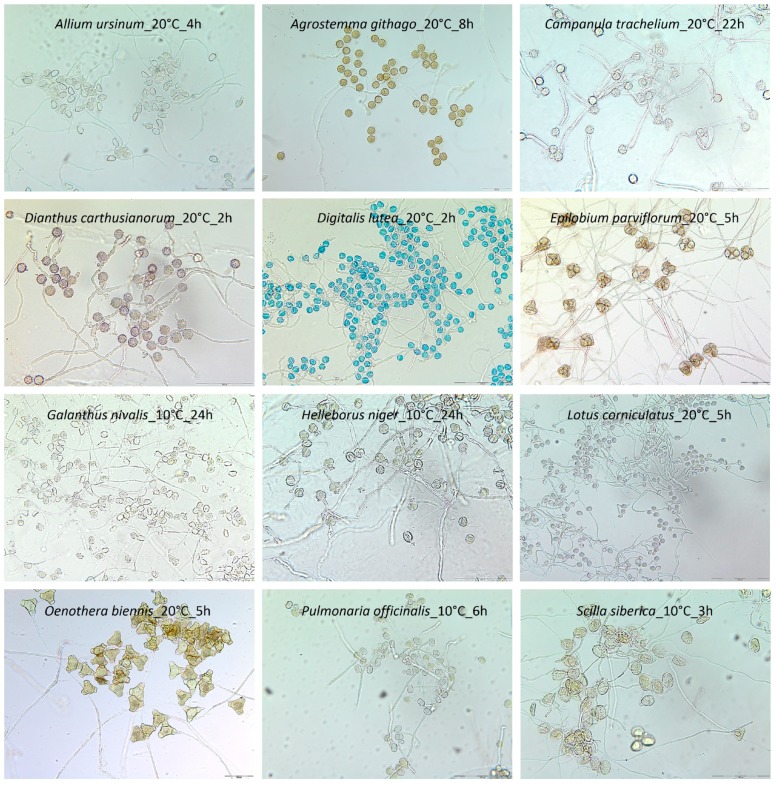
In vitro pollen germination and pollen tube growth in the study species in alphabetical order. Pictures show the appearance in the optimum temperature range for germination after pollen tube growth had been stopped with glycerol. Scale = 200 µm.

There was a clear relationship between the lower temperature limit for pollen germination and the flowering month of a plant species (Figure 1a; *p* < 0.001, Spearman) and the mean minimum air temperature in the coldest flowering month (Figure 1b; *p* = 0.001, Spearman). Pollen grains of winter and early spring flowering plants (January–March) still germinated below 0 °C, those of spring flowering plants (April–May) started to germinate around zero, and those of summer flowering species between 2 °C (*Epilobium*, *Digitalis*) and 5 °C (the remaining species) Figure 2. Furthermore, pollen of winter and early spring flowering plants showed high relative germination rates already around zero whereas pollen of most summer flowering species germinated poorly in the cold compared to the species specific mean control values determined in the optimum temperature range (10 °C–20 °C). For comparability among species, germination values in Figure 2 are given in percent of the respective control value (=100%) in the optimum temperature range. The appearance of the pollen samples during in vitro germination and tube growth in the optimum temperature range is shown in Figure 3.

**Figure 4:**
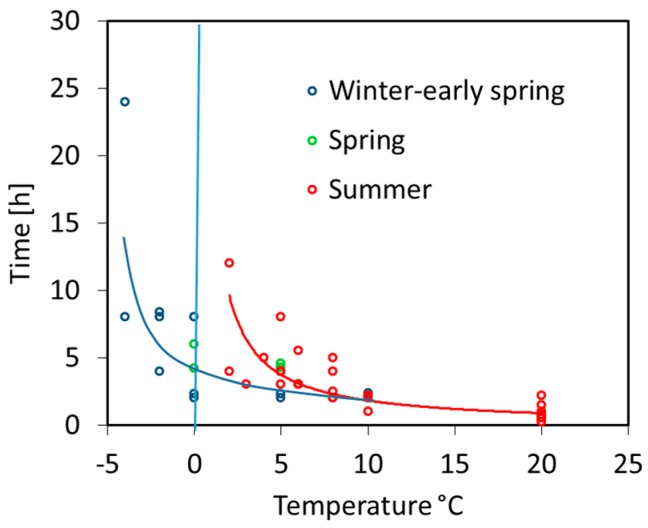
Delay of the germination start in hours depending on temperature for the different phenotypes. Trend lines show the power trendline for the increase in lag time with decreasing temperatures in summer flowering species (red, *y* = 19.8*x*^−1.04^) and winter and spring flowering species (blue, *y* = 13.5*x*^−0.76^).

Upon application of pollen grains on the agarose medium, germination started with a different delay depending on temperature and species (Figure 4). Between 10 and 20 °C pollen germination began within 1–2 h or less (0.5 h in *Epilobium* at 20 °C). Below 10 °C, summer flowering species needed increasingly longer: the delay was up to 8 h at 5 °C and up to 12 h in species which still germinated at 2 °C. At zero, winter and spring flowering species germinated on average about as rapidly as summer flowering species at 5 °C with *Scilla* and *Helleborus* showing the shortest lag time of 2 h only. Below zero only pollen of winter flowering species germinated: within 4 h (*Helleborus*) and 8 h (*Galanthus, Scilla*) at −2 °C, and within 8 h (*Scilla*) and 24 h (*Galanthus*) at −4 °C.

**Figure 5:**
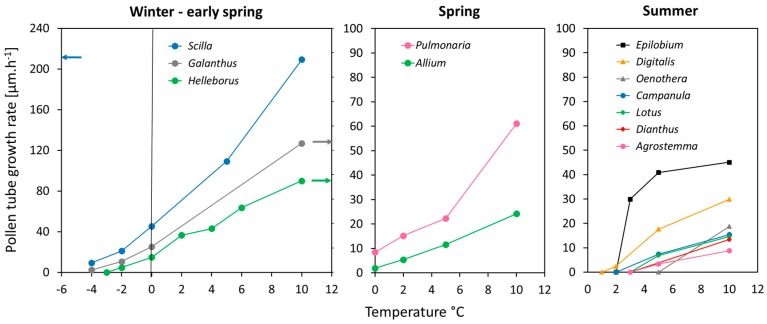
Mean pollen tube growth rates of the different phenotypes in dependence of temperature. Arrows in the diagram for winter and early spring species mark the affiliation of the data set to the left (*Scilla*) and right (*Galanthus*, *Helleborus*) *y*-axis.

Pollen tube growth was generally faster in winter and spring flowering species than in summer-flowering species at the same temperature (Figure 5), but differed greatly among species within the same phenogroup. Cluster analysis for the mean growth rates at 10, 5, and 0 °C as variables resulted in four distinct groups: group 1 included only *Scilla* which showed an exceptionally fast pollen tube growth already below zero; group 2 comprised of *Galanthus* (winter-early spring), *Pulmonaria* (spring), and *Epilobium* (summer); group 3 comprised of *Helleborus* (winter), *Allium* (spring), and *Digitalis* (summer), and group 4 was comprised of the remaining summer flowering species.

**Figure 6:**
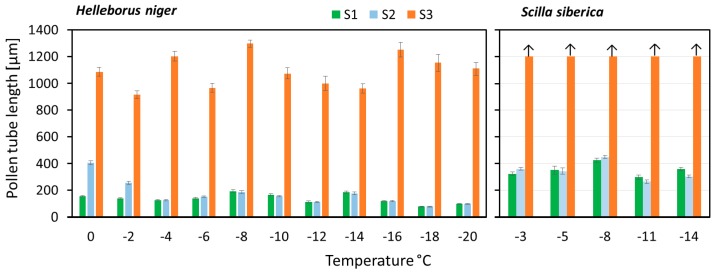
Frost after-effects on pollen tube growth in the winter flowering *Helleborus niger* and the early spring flowering *Scilla siberica*. Bars show the mean pollen tube lengths (± SE) at the end of the cooling down phase (S1), the frost phase (S2), and the 24 h recovery phase (S3) in the step cooling experiment with different target temperatures. Arrows: Pollen tubes were too long to be measured.

Pollen germination and pollen tube growth stopped at a species specific low temperature limit but continued after rewarming as long as the male gametophyte was not irreversibly impaired. There were marked differences between phenological types up to which temperature recovery was possible. Activated male gametophytes of the winter flowering *Helleborus* and the early spring flowering *Scilla* survived severe frost without visible functional disorder (Figure 6). In *Helleborus*, pollen tube growth continued in the constant warm phase even after an exposure to −20 °C. As the germination rate was already at its maximum after the cooling down phase it might be assumed that already germinated pollen resumed growth after the cooling phase. For *Scilla*, recovery of pollen tube growth was observed down to −14 °C but might also occur after exposure to lower temperatures (not measured). As pollen tubes of *Scilla* grow fast, pollen tubes were too long to be measured at the end of the recovery phase. 

**Figure 7:**
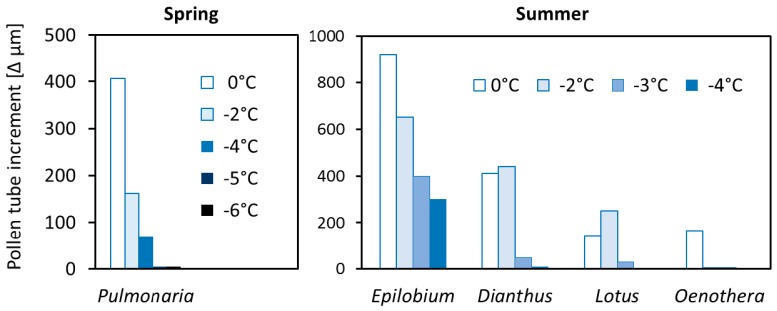
Frost after-effects on pollen tube growth in the spring flowering species *Pulmonaria* and in four summer-flowering species. Bars show the difference between the mean pollen tube lengths at the end of the 24 h recovery phase (S3) and the end of the cooling down phase (S1) in the step cooling experiment with different target temperatures.

Male gametophytes of the spring flowering *Pulmonaria* and of the tested summer-flowering species lost functionality more or less rapidly below zero (Figure 7). In all species, pollen germination was saturated during the cooling down phase. Therefore, it can be assumed that growing pollen tubes were damaged by frost and did not grow further after warming up. Germinating pollen of *Oenothera* was particularly frost sensitive. Gametophytes of *Lotus* and *Dianthus* did not recover below −2 °C. Pollen tubes of *Epilobium* grew further after the frost exposure, but the increment decreased with the treatment temperature.

## 3. Discussion

There is a clear relationship between the performance of the male gametophyte in the cold and the flowering time. For most summer flowering species, the cold limit for pollen germination and pollen tube growth was between 5 and 10 °C, which is in the range of what has been reported by Rosbakh and Poschlod [13] for lowland plants in Central Europe. Pollen of only a few species (*Digitalis lutea*, *Epilobium parviflorum*) still germinated at 2 °C. In spring flowering species, pollen germination and pollen tube growth stopped just below 0 °C; in winter flowering and early-spring flowering species, however, pollen germination stopped between −2 und −4 °C. This is a remarkable finding, since even cold-adapted higher plants do not grow at sub-zero temperatures [30,31], though metabolic processes such as photosynthesis and respiration still occur below zero [32]. Complex growing activities, however, such as cell division, cell elongation, and differentiation do not take place, mainly because sink activity (i.e., the utilization of carbohydrates) is constrained in the cold [33]. Another key factor for cell growth is the cytoskeleton, which disintegrates or changes the conformation under suboptimal temperatures [34,35]. In contrast to higher plants, individual cells and less complex cell systems of cold adapted organisms can grow and propagate even at temperatures below the freezing point. Examples are cryophilic bacteria which can still multiply at −15 °C [36], Antarctic diatoms [37], and fungi such as snow molds which still show mycelial growth at −3 °C [38]. These organisms have evolved cold-adaptive mechanisms, such as cold-active enzymes, altered membrane lipid conformation, and antifreeze proteins, which enable cell division, cell enlargement, and cell wall construction to occur at sub-zero temperatures [36,39]. Similarly, male gametophytes in flowering plants can be regarded as small semi-autonomous organisms of low complexity. A pollen tube is in principle a single cell (apart from the sperm cells inside) that puts all its metabolic energy into cell wall construction at the tip. The protoplast itself hardly enlarges, but travels with the pollen tube tip, closing the vacated space behind with callose plugs. It is conceivable that because of the low organization level male gametophytes are more flexible in the adaptation to frost than the sporophyte as a whole. 

The speed of imbibition and metabolic activation of the ripe male gametophyte depends on the water content at the time of dispersal and on the inflow of water from the stigma upon pollination (reviewed in [40]). With regard to the water content, Nepi et al. [28] and Franchi et al. [29] classified pollen of different plant taxa as partially dehydrated (PD; water content below 30%) and partially hydrated (PH; water content higher than 30%). On the stigma, PH pollen germinates within a few minutes to less than an hour, whereas PD pollen starts later than 60 min because pollen rehydration needs more time [41]. Though pollen activation on the artificial medium used in the present study was possibly faster than it would be in vivo, the lag time for the different species corresponded largely to the pollen water content type (for classification see Table 1): at 20 °C, germination delay was 1–2 h in PD pollen, and 15 min to 1 h in PH pollen. At lower temperatures the germination delay, as well as pollen tube growth rates, clearly reflected the adaptation of the metabolism to the different thermal climates during the flowering period (see Table 1). At 10 °C, pollen still germinated within 1–2 h in all phenogroups. Below that temperature, the lag time steadily increased in summer flowering species. In spring flowering species the increase in lag time began at +5 °C and in winter flowering species at zero. The differences among phenotypes were even more pronounced in the speed of pollen tube growth: pollen tubes of summer flowering species grew at 10 °C as slowly as in winter and spring flowering plants at zero. Single species within the phenogroups showed a different activity pattern as would have been expected from the flowering time. With regard to the pollen performance at low temperatures, *Epilobium* and *Digitalis* rather fitted into the cluster group of winter and spring-flowering species than in the group of summer flowering species, which could be seen as adaptation to the broad altitudinal distribution from the lowlands to the alpine and—for *Epilobium*—the long flowering time from summer until the cooler autumn. The male gametophyte of *Scilla siberica,* a species which is native to southwestern Russia, turned out to be exceptionally well adapted to the cold: the mean growth rate was already 50 µm·h^−1^ at 0 °C, and still 10 µm·h^−1^ at −4 °C. Such high speeds around zero have been found in some high-mountain plants so far [9]. 

Generally, plant tissues in an active metabolic state do not, or only to a limited extent, survive frost [42]. Plants from cold environments, such as high mountain plants, can tolerate the formation of extracellular ice and subsequent freeze-dehydration to a certain degree, whereas intracellular ice formation is lethal at any time [43,44,45]. With few exceptions, inactive pollen grains lack vacuoles [46]. PD pollen at its dispersal is usually desiccation-tolerant [47], and, after further drying under controlled conditions, may even tolerate deep freezing in liquid nitrogen for the purpose of cryopreservation in pollen banks [48]. Conversely, PH pollen is highly susceptible to desiccation [47], and thus to freezing [29]. During pollen rehydration and early germination new vacuoles form [49]. Later, during pollen tube growth, numerous small vacuoles appear in the nuclear zone and a single or few larger vacuole(s) appear in the vacuolar zone at the proximal end of the pollen tube cell [50]. Against this background, we expected that PH pollen and PD pollen after rehydration, and even more during germination and pollen tube growth, would not survive subzero temperatures. In the step-cooling experiment, we tested the recovery capacity after the frost treatment. Resumption of pollen tube growth, or at least continued pollen germination, indicated survival of the male gametophyte. Our hypothesis was confirmed for the male gametophyte of the investigated summer flowering and spring-flowering species which suffered irreversible damage already from mild sub-zero temperatures. Male gametophytes of winter and early-spring flowering species, however, were exceptionally frost tolerant and survived the exposure of 12 h at −14°C (*Scilla*) and temperatures below (*Helleborus*). Remarkably, pollen of most frost sensitive species is of the PH type, whereas pollen of the frost tolerant species belongs to the PD type. We do not know whether, and if so at which freezing temperatures, ice forms within the male gametophytes. It is conceivable that hydrated pollen and pollen tubes supercool for a certain period before ice nucleation takes place. As outlined above, living cells survive only extracellular ice, which in the case of pollen tubes might form in the free space of the pollen tube adjacent to the protoplast. However, extracellular freezing leads to increasing freeze-dehydration of the protoplast, which is tolerated by plant cells to a variable extent. Pollen tube cells of frost tolerant plant species obviously are highly resistant to extracellular freezing and freeze-dehydration. Possible reasons for a high desiccation tolerance are protective sugars which accumulate in PD pollen during pollen maturation [4,40,51,52] and might also be present during germination and pollen tube growth. For *Helleborus* pollen, Vesprini et al. [53] could show that a decrease in temperature led to an inter-conversion of polysaccharides to sucrose and monosaccharides. Hydrolysis of complex sugars is a general strategy of plant organs to adjust to freezing temperatures [42]. Other mechanisms to stabilize cell structures during desiccation are LEA (late embryogenesis abundant) and heat stress proteins, which could also be detected in mature pollen grains (reviewed in [40]). Further research is needed to elucidate the mechanisms of ice nucleation and dehydration tolerance in male gametophytes during germination and tube growth, a topic which would contribute to the general understanding of freeze avoidance in plants [54]. 

Low temperature limits of pollen performance can serve as a rough predictor for plant distribution along latitudinal and elevational temperature gradients, and may indicate adaptation to the seasonal temperature conditions ([13], present study). In detail, however, it remains open whether the in vivo performance of the male gametophyte is the same as in vitro. The functional temperature limits of the female tissues which control, nourish, and guide the growing pollen tubes via a variety of signals [55] may be different. Though methodically much more challenging, it would also need information about female functions and the male-female interplay in the cold for drawing conclusions about cold limits of sexual processes in a plant species. 

Another question is whether the cessation of male and female functions at low temperatures is transient or irreversible—with differing consequences for the reproductive outcome. A transient arrest (e.g., during cool nights) must not automatically lead to a failure of reproductive processes, but gives the opportunity for further development as soon as temperatures rise again, as shown for high-mountain plants [9]. Only in the case of irreversible functional impairment are the cold limits absolute. In the male gametophytes of plant species studied here, the transient and absolute cold limit were mostly at different temperatures. In summer-flowering species, male growth stopped above zero but resumed when transferred to warm temperatures again (data not shown), indicating a temporary functional limit. The absolute limit for male growth was reached some degrees lower at sub-zero temperatures at which the capacity to recover rapidly decreased. For the male gametophytes of *Pulmonaria*—the only spring flowering species that was tested at freezing temperatures—the transient and the absolute functional limit laid close together (around −2 °C). By contrast, in the winter and early spring flowering *Helleborus* and *Scilla*, the discrepancy between transient and absolute limit was considerable: the transient limit when pollen germination and pollen tube growth stopped was between −3°C (*Helleborus*) and around −5 °C (*Scilla*), but the capacity to recover remained intact even after exposure to severe frost. 

At temperatures below zero, apart from functional limits, the freezing tolerance of all reproductive structures plays an essential role [44,45]. Summer-flowering lowland species are not freezing tolerant, and are damaged as soon as ice forms within the tissues, which happens just a few degrees below zero [42]. In summer phenotypes, male and female functions cease more or less at the same low temperature in close proximity to the temperature at which frost damage occurs. In winter and early spring flowering plants, however, the damage thresholds for male and female structures lie far apart. In *Helleborus* for instance, carpels and ovules lose their functions when they are frost damaged around −10 °C (Johanna Wagner and Martin Kogler, unpublished results), whereas germinating male gametophytes are still alive after exposure to −20 °C. This example shows that on the basis of the frost tolerance of the male gametophyte alone the temperature limits for the reproductive functionality would be unrealistic. Thus, for an overall assessment of the reproductive limits of a plant it is essential to determine both the functional cold limits and the cold/freezing tolerance for all reproductive structures. 

To summarize, the study revealed a clear relationship between pollen performance in the cold and the minimum air temperatures in the flowering period. Cold tolerance differed among species within a phenological group anticipating differences in the adaptive potential to temperature fluctuations. Furthermore, we could show that male gametophytes of cold-adapted plant species show a high degree of frost tolerance during germination and tube growth which can be higher than in the plant as a whole. It has still to be clarified whether the cellular mechanisms that enable pollen tubes to grow at sub-zero temperatures and to tolerate frost are similar to those in cryophilic organisms.

## 4. Materials and Methods

### 4.1. Study Species

The study was carried out on common herbaceous lowland species flowering in winter and early-spring (*Galanthus nivalis*, *Helleborus niger*, *Scilla siberica*), in spring (*Pulmonaria officinalis*, *Allium ursinum*) and in summer (*Agrostemma githago*, *Campanula trachelium*, *Epilobium parviflorum*, *Digitalis lutea*, *Dianthus carthusianorum*, *Lotus corniculatus*, and *Oenothera biennis*). For more details see Table 1. Most study species are widely distributed in Middle Europe, with a core distribution in the colline and montane vegetation belt. To avoid effects of pollen age, only pollen from freshly opened anthers was used. Flowers were collected in the morning of the day of the experiment either in the Botanical Garden of Innsbruck or in natural stands in the surroundings of Innsbruck (47°16′ N, 11°24′ O) between about 600 and 800 m above sea level. 

### 4.2. Medium and Test System for Pollen Germination

Pollen germination and tube growth were checked in vitro on a solidified germination medium according to Boavida and McCormick [26]. The double concentrated medium (0.02% H_3_BO_3_, 10 mM CaCl_2_·2H_2_O, 10 mM KCl, 2 mM MgSO_4_·7H_2_O, 3% low melt Agarose) adjusted to pH 7.5 using 0.1 N NaOH, was aliquoted á 500 µL into Eppendorf tubes and stored in a freezer until further usage. To prepare the final medium, the medium was liquefied at 65 °C in a block heater. Then, 400 µL of a 2.5-fold sucrose solution and 100 µL distilled water were added and vortexed. The optimum sucrose concentration determined in a pre-test was 10% for all species. To guarantee preparations with a constant medium thickness and to get several parallels in a small space, medium pads of the same size were made. With a pipette (blue tips which do not clog so easily) three groups of six small medium droplets were applied to the flat depressions in the lid of 48-multiwell plates. Immediately after a broad glass slide (38 × 76 mm) was laid on each droplet group. After solidification in the fridge, the slides with the attaching medium-pads could be removed using a scalpel. Each slide (later referred to as sample S) was immediately transferred to a petri dish lined with a wet filter paper disc and a coarse plastic grid to avoid wetting of the slide. Slides with medium pads in Petri dishes could be stored up to five days in the fridge in a zipper plastic bag. The petri dish system allowed for the easy handling of individual pollen samples, and—which is most important—a fast adjustment to the treatment temperature within few minutes. 

### 4.3. Pollen Application

Depending on the species and the pollen quantity per flower, pollen was harvested in different ways (for details see Table 1): (1) open anthers of a number of flowers from different individuals were mixed in Eppendorf tubes and pollen was applied with an interdental microbrush (DenTek, Maryville, TN, USA) onto the medium pads; (2) in the case of small flowers, whole flowers were dabbed onto the medium. Pollinated slides were enclosed in precooled Petri dishes; dishes were sealed with Parafilm and immediately exposed to the test temperature. 

### 4.4. Temperature Treatments

For temperatures down to −8 °C a programmable temperature incubator (Binder KB 53, Tuttlingen, Germany) was used. Experiments at lower temperatures took place in temperature-controlled chest freezers (Liebherr, Lienz, Austria). The temperature inside the freezing compartment is controlled by a special software (LabView 2012, National Instruments Corporation, Austin, TX, USA; programmed by Othmar Buchner) that realizes user-defined cooling profiles with a temperature accuracy of ±0.2 Kelvin (K). Temperature control is put into effect by targeted heating against the permanently cooling chest freezer by use of cabin heaters (Nimbus B200, DBK Rülzheim, Germany). Temperature equalization within the freezing compartment is managed by ventilators. 

**Figure 8:**
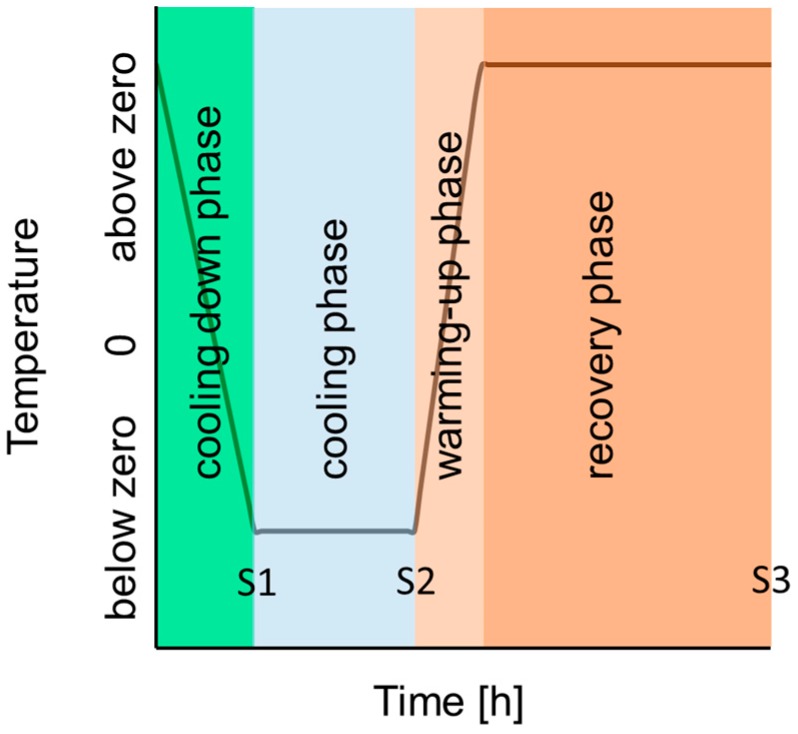
Scheme of the temperature profile during step cooling. S1, S2, and S3 mark the sampling times.

To determine germination rates and the velocity of pollen tube growth, several samples were exposed in parallel at constant temperatures. To determine the lag time of germination, samples were microscopically checked at 15–30 min intervals. After germination had started, samples were taken at 1–2 h intervals up to 24 h, and pollen tube growth was stopped with glycerol (see below). To test for after-effects of different low temperatures on pollen performance, pollen of a part of the study species was subjected to step cooling (Figure 8). The first three sections of the temperature profile were programmed following natural temperature profiles on days with night frosts, and consisted of a cooling down phase, a cooling phase, and a warming-up phase (2–3 K·h^−1^). The subsequent recovery phase at warm temperatures for another 24 h should give information about the recovery ability of pollen tube growth. The starting temperatures and the cooling down rate were adjusted to the velocity of pollen germination and pollen tube growth and determined for each species in a pretest. At the beginning of the cooling phase, germination should be largely completed and pollen tubes should not be longer that 200 µm. The cooling down rate was 2 K·h^−1^ in all species, except for Scilla (3 K·h^−1^). The starting temperatures were 10 °C for *Helleborus* and *Scilla*, 12 °C for *Pulmonaria*, 14 °C for *Epilobium* and *Oenothera*, and 16 °C for *Dianthus* and *Lotus*. Target temperatures during the cooling phase were set in 1–3 K steps and applied for 12 h (*Helleborus*), 8 h (*Scilla*, *Pulmonaria*), and 4 h (summer flowering species). Samples were taken at the end of the cooling-down phase (sample S1), at the end of the cooling phase (S2, only *Helleborus* and *Scilla*), and at the end of the recovery phase (S3). Pollen tube growth was stopped by adding a drop of glycerol (86%, Rotipuran, Carl Roth, Germany) on each agarose-pad and covering all pads together with a large cover slip (24 × 50 mm). Pollen samples covered with glycerol can be stored in a horizontal position for at least one year.

### 4.5. Assessment of Pollen Germination and Pollen Tube Growth

Pollen germination counts were made at random in five fields per pad, which gave a total of 30 counts per sample. A pollen grain was classified as germinated if the length of the pollen tube was equal to or greater than the diameter of the pollen grain. Additionally, the lengths of the longest five pollen tubes per pad were measured, which again gave a total of 30 measurements per sample. Evaluation was made under a microscope with differential interference contrast at 10–40× magnification using an image analysis system (Cell^D, Olympus). Samples from temperature step cooling were evaluated as follows: for spring and summer flowering species, whose pollen tubes did not grow below zero, the differences in pollen tube lengths between the sampling times S3 and S1 were calculated; for *Helleborus* and *Scilla* the absolute average lengths for the sampling times S1, S2, and S3 are presented, because pollen tubes grew further during the cooling phase and, in the case of *Scilla*, were too long to be measured at the end of the recovery phase. 

### 4.6. Statistics

Spearman’s rank-order correlation was used to analyze the relationship between the low temperature limit for pollen germination and the month of flowering and the mean minimum air temperature in the coldest month during which a species flowers. The percentage of pollen germination is species specific and therefore not directly comparable; to compare germination ability at different temperatures among species, germination values were related to the mean maximum value (=100%) determined in the optimum temperature range for each species. Cluster analysis was performed to group species according to their dynamics of pollen tube growth at different temperatures. All analyses were carried out at the significance level α = 0.05 using the statistical package IBM SPSS Statistics 23 (IBM, New York, NY, USA). 

## Figures and Tables

**Table 1:** List of investigated species with details on the pollen hydration status at dispersal, the flowering time, the mean minimum temperature and, in square brackets, the absolute minimum temperature in the coldest month of the respective flowering period.

Plant Species	Family	Hydration Status ^1^	Flowering Season	Flowering Months	Minimum Air Temperature [°C] ^2^	Number of Individuals/Flowers Per Test	Pollen Application
*Helleborus niger* L.	Ranunculacae	PD	Winter	12–3	−4 (−21.1)	5/5	mixture
*Galanthus nivalis* L.	Amaryllidaceae	PD	Winter-early spring	1–3	−4 (−21.1)	10/10	mixture
*Scilla siberica* HAW.	Asparagaceae	PD	Early spring	2–3	−2.8 (−15.1)	10/10	mixture
*Pulmonaria officinalis* L.	Boraginaceae	PD	Spring	3–4	1.0 (−15.1)	5-–10/10–20	single flowers
*Allium ursinum* L.	Amaryllidaceae	PD	Spring	4–5	4.7 (−7.0)	5–10/15–20	mixture
*Epilobium parviflorum S*CHREB.	Onagraceae	PH	Summer	7–9	10.2 (−0.3)	5/15–20	single flowers
*Agrostemma githago* L.	Caryophyllaceae	PH	Summer	6–7	12 (3.9)	5/5–10	single stamens
*Dianthus carthusianorum* L.	Caryophyllaceae	PH	Summer	6–8	12 (3.9)	5/5	single stamens
*Digitalis lutea* L.	Plantaginaceae	PD	Summer	6–7	12 (3.9)	5/10	mixture
*Lotus corniculatus* L.	Fabaceae	PD	Summer	6–8	12 (3.9)	5–10/5–10	mixture
*Oenothera biennis* L.	Onagraceae	PH	Summer	6–8	12 (3.9)	5–10/5–10	mixture
*Campanula trachelium* L.	Campanulaceae	PH	Summer	7–8	13.6 (4.4)	5/5	mixture

^1^ Hydration status based on pollen shape and size according to [28,29]; PD, partly dehydrated pollen; PH, partly hydrated pollen; ^2^ Long-term air temperature at 2 m height (1981–2010) in Innsbruck (578 m above sea level), provided by the Central Institute for Meteorology and Geodynamics in Austria.
